# Efficacy of European starling control to reduce *Salmonella enterica *contamination in a concentrated animal feeding operation in the Texas panhandle

**DOI:** 10.1186/1746-6148-7-9

**Published:** 2011-02-16

**Authors:** James C Carlson, Richard M Engeman, Doreene R Hyatt, Rickey L Gilliland, Thomas J DeLiberto, Larry Clark, Michael J Bodenchuk, George M Linz

**Affiliations:** 1 U.S. Department of Agriculture, Animal and Plant Health Inspection Service, Wildlife Services, National Wildlife Research Center, 4101 LaPorte Avenue, Fort Collins, CO 80521, USA; 2 College of Veterinary Medicine and Biomedical Sciences, Colorado State University, Diagnostic Laboratories, Bacteriology Section, Fort Collins, CO 80523-1644, USA; 3 U.S. Department of Agriculture, Animal and Plant Health Inspection Service, Texas Wildlife Services, 5730 Northwest Parkway, Suite 700, San Antonio, TX 78249, USA; 4 U.S. Department of Agriculture, Animal and Plant Health Inspection Service, Wildlife Services, National Wildlife Disease Program, 4101 LaPorte Avenue, Fort Collins, CO 80521, USA; 5 U.S. Department of Agriculture, Animal and Plant Health Inspection Service, Wildlife Services, National Wildlife Research Center, 2110 Miriam Circle, Suite B, Bismarck, ND 58501-2502, USA

## Abstract

**Background:**

European starlings (*Sturnus vulgaris*) are an invasive bird species known to cause damage to plant and animal agriculture. New evidence suggests starlings may also contribute to the maintenance and spread of diseases within livestock facilities. Identifying and mitigating the risk pathways that contribute to disease in livestock is necessary to reduce production losses and contamination of human food products. To better understand the impact starlings have on disease transmission to cattle we assessed the efficacy of starling control as a tool to reduce *Salmonella enterica *within a concentrated animal feeding operation. We matched a large facility, slated for operational control using DRC-1339 (3-chloro-4-methylaniline hydrochloride, also 3-chloro p-toluidine hydrochloride, 3-chloro-4-methylaniline), with a comparable reference facility that was not controlling birds. In both facilities, we sampled cattle feed, cattle water and cattle feces for *S. enterica *before and after starling control operations.

**Results:**

Within the starling-controlled CAFO, detections of *S. enterica *contamination disappeared from feed bunks and substantially declined within water troughs following starling control operations. Within the reference facility, detections of *S. enterica *contamination increased substantially within feed bunks and water troughs. Starling control was not observed to reduce prevalence of *S. enterica *in the cattle herd. Following starling control operations, herd prevalence of *S. enterica *increased on the reference facility but herd prevalence of *S. enterica *on the starling-controlled CAFO stayed at pretreatment levels.

**Conclusions:**

Within the starling-controlled facility detections of *S. enterica *disappeared from feed bunks and substantially declined within water troughs following control operations. Since cattle feed and water are obvious routes for the ingestion of *S. enterica*, starling control shows promise as a tool to help livestock producers manage disease. Yet, we do not believe starling control should be used as a stand alone tool to reduce *S. enterica *infections. Rather starling control could be used as part of a comprehensive disease management plan for concentrated animal feeding operations.

## Background

Concentrated animal feeding operations (CAFO's) are sources for new, more infectious or antibiotic resistant microorganisms that can spread to humans and the environment [1]. For example, feeder cattle raised in CAFO's have been linked to the contamination of ground beef with antibiotic resistant *S. enterica *[2]. This is not an isolated problem, virtually all CAFO's within the U. S. experience chronic problems with livestock diseases [3] and domestic cattle (*Bos taurus*) are known reservoirs of many gastrointestinal (GI) pathogens including the bacterium *Salmonella enterica *[4,5]. Because of economic losses and human health risks managing disease in CAFO's is of paramount importance to livestock producers. To manage disease, producers need better information on the specific risk pathways that contribute to the spread and maintenance of pathogenic microorganisms within their CAFO's. One of these risk pathways is the wildlife-livestock interface.

*Salmonella enterica *is a ubiquitous microorganism in CAFO's that has been linked to peridomestic wildlife use of feedlots and dairies [6,7]. In CAFO's, cattle typically acquire *S. enterica *from other infected livestock which spread the pathogen throughout the herd via contaminated cattle feces [8], cattle feed [9], and water [10]. Reducing contamination from these sources is important because clinical and subclinical *S. enterica *infections in cattle can cause significant economic losses to producers and can lead to carcass contamination at the slaughterhouse [5,11]. Carcass contamination contributes to human salmonellosis, which is responsible for an estimated 1.3 million human cases, 15,600 hospitalizations, and 550 deaths each year [12]. Recent empirical evidence suggests that small mammal and bird feces may be a significant source of *S. enterica *contamination of animal feed, which by itself is capable of explaining the infection levels seen in cattle herds [6].

European starlings (*Sturnus vulgaris*) were placed on the Invasive Species Specialist Groups list of "100 Worlds Worst" biological invaders [13]. Starlings damage plant and animal agriculture by consuming crops destined for human and livestock consumption [14,15]. Starling damage to agriculture within the U.S. was estimated at $800 million annually [16]. Feed consumption by starlings in CAFO's is well documented. Besser et al. [14] reported that individual starlings in cage trials consumed approximately 1 ounce of livestock ration per day. Glahn and Otis [17] reported consumption of about 10.5 lbs of cattle feed per 1,000 bird minutes. White et al. [18] estimated that starlings consumed 35 metric tons of corn from feedlots within their study area around Milan, Tennessee between 1976 and 1977.

In addition to crop damage and livestock feed consumption starlings are known carriers of many human and cattle pathogens, including *S. enterica *[19-22]. Starlings have also been implicated as a source for *S. enterica *contamination of cattle feed and water [23] and this information was collected, in part, from the two CAFOs used for this study. Additionally, many other publications suggest wild birds may contribute to the maintenance and spread of *S. enterica *[5,6,10,24,25]. Currently, there is no data assessing starling control as a tool to reduce the amplification and spread of *S. enterica *within CAFO's.

Population management programs have been implemented in many parts of the world to mitigate bird damage to agriculture [14,26,27]. Within the United States a common form of starling management involves lethal chemical control using DRC-1339 (3-chloro-4-methylaniline hydrochloride, also 3-chloro p-toluidine hydrochloride, 3-chloro-4-methylaniline). It is a slow-acting toxicant used to control starlings and blackbirds [21,27]. DRC-1339 is effective for reducing numbers of starlings in livestock facilities. Besser et al. [14] reduced a starling population by about 75% after spreading 1% DRC 1339-treated poultry pellets at a cattle feedlot in Nevada. West [28] reduced a roost of 250,000 starlings in Colorado 60% by baiting feedlots and pastures.

Our objective was to assess the efficacy of starling control as a potential tool to reduce *S. enterica *within CAFO's. We matched a large CAFO in the Texas panhandle, slated for operational control using DRC-1339, with a comparable reference facility that was not controlling birds. We sampled cattle feed, cattle water troughs and fresh cattle fecal pats within pen lanes for *S. enterica *before and after starling control operations in both the starling-controlled and reference facilities. Specifically, we wanted to know if the starling-controlled CAFO experienced reduced *S. enterica *contamination within cattle water troughs, feed bunks and cattle feces relative to the reference facility.

## Results

### Efficacy of starling control operations

**Figure 1:**
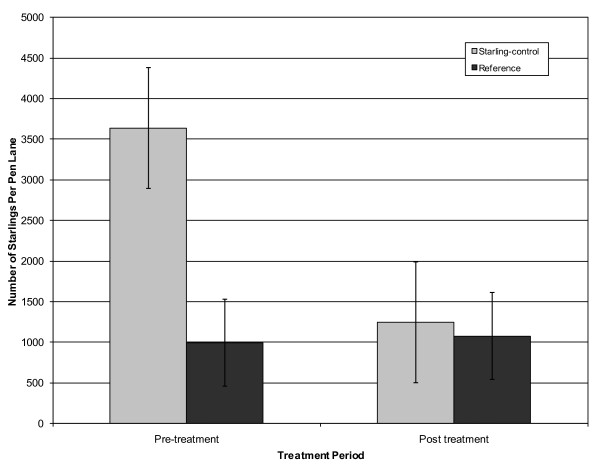
**Estimated number of European starling within pen lanes**. The mean number and standard deviation of European starling estimates from the starling-controlled and reference CAFO's during pre and post DRC-1339 starling control periods. All estimates were taken from 2 CAFO's located in Moore County, Texas from 18 January through 18 February 2010.

DRC-1339 control operations were effective at reducing the number of starlings present within pen lanes (*F*_1, 8 _= 30.64, *P *= 0.0006). On the starling-controlled CAFO there were on average 3588 (95% CI = 2895, 4280) starlings within pen lanes before DRC-1339 control and 1246 (95% CI = 554, 1939) starlings within pen lanes after DRC-1339 control. On the reference CAFO there were on average 996 (95% CI = 303, 1688) starlings within pen lanes before DRC-1339 control and 1054 (95% CI = 361, 1746) starlings within pen lanes after DRC-1339 control. On the starling-controlled CAFO, mean starling numbers per pen lanes were reduced 65.7% following DRC-1339 starling control operations (Figure 1).

### Contamination within water troughs, feed bunks and cattle fecal samples

**Figure 2:**
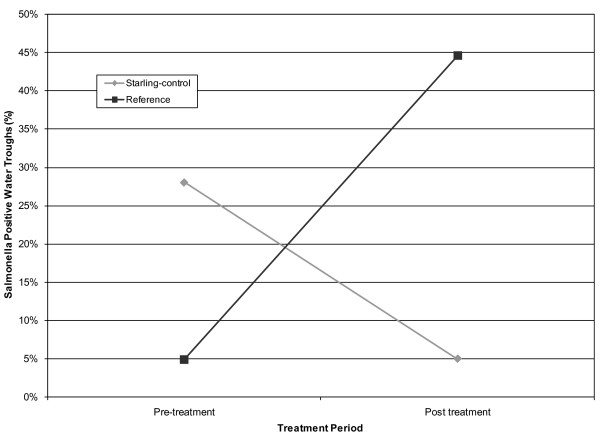
***Salmonella *contamination within cattle water troughs**. The percentage of cattle water troughs testing positive on the starling-controlled and reference CAFO's during pre and post DRC-1339 starling control periods. All samples were collected from 2 CAFO's located in Moore County, Texas from 18 January through 18 February 2010.

Starling control was associated with decreased *S. enterica *contamination in water troughs (*F*_1, 8 _= 30.64, *P *< 0.0001, Figure 2). On the treatment CAFO 28% of water troughs were contaminated with *S. enterica *before DRC-1339 control (95% CI = 18%, 38%) and 5% of water troughs were contaminated after DRC-1339 control (95% = CI 0%, 15%). On the reference CAFO 5% of water troughs were contaminated before DRC-1339 control (95% CI = 0%, 15%) and 45% of water troughs were contaminated after DRC-1339 control (95% CI = 34%, 55%).

**Figure 3:**
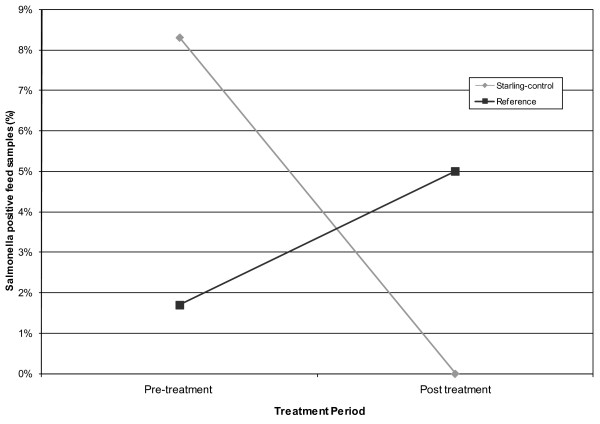
***Salmonella *contamination within cattle feed bunks**. The percentage of cattle feed samples testing positive on the starling-controlled and reference CAFO's during pre and post DRC-1339 starling control periods. All samples were collected from 2 CAFO's located in Moore County, Texas from 18 January through 18 February 2010.

Starling control was associated with decreased *S. enterica *contamination within feed bunks (*F*_1, 16 _= 3.27, *P *= 0.0895, Figure 3). On the treatment CAFO 8% of feed bunks were contaminated with *S. enterica *before DRC-1339 control (95% CI = 1%, 15%) and 0% of feed bunks were contaminated after DRC-1339 control (95% = CI 0%, 7%). On the reference CAFO 2% of feed bunks were contaminated before DRC-1339 control (95% CI = 0%, 8%) and 5% of feed bunks were contaminated after DRC-1339 control (0%, 12%).

**Figure 4:**
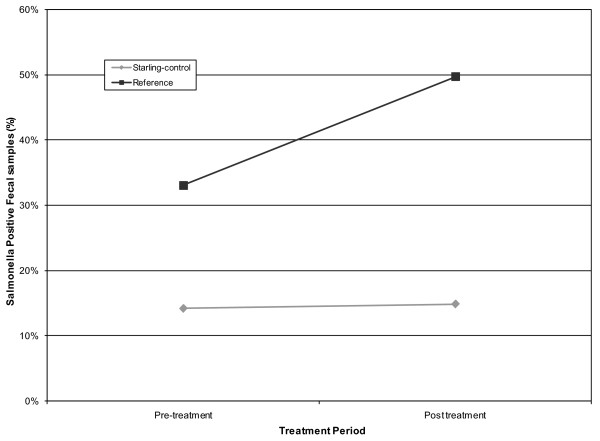
***Salmonella *contamination within cattle fecal samples**. The percentage of cattle fecal samples testing positive on the starling-controlled and reference CAFO's during pre and post DRC-1339 starling control periods. All samples were collected from 2 CAFO's located in Moore County, Texas from 18 January through 18 February 2010.

Starling control was not observed to reduce prevalence of *S. enterica *in the cattle herd (*F*_1, 16 _= 1.31, *P *= 0.2688, Figure 4). On the starling-controlled CAFO 14% of fecal samples were contaminated with *S. enterica *before DRC-1339 control (95% CI = 0%, 29%) and 15% of fecal samples were contaminated after DRC-1339 control (95% = CI 0%, 30%). On the reference CAFO 33% of fecal samples were contaminated before DRC-1339 control (95% CI = 18%, 48%) and 50% of fecal samples were contaminated after DRC-1339 control (35%, 64%).

### Serogroup and serotype data

From all sample types within both CAFO's we isolated five different serogroups, with C1 (46%) and E (39%) comprising the majority serogroups isolated (Table 1). Comparisons between CAFO's suggested there was little difference in the serogroups isolated. We isolated three *Salmonella *serogroups from cattle feces within the starling controlled CAFO with E (50%) and C1 (28%) comprising the majority of the cattle fecal isolates. We isolated three *Salmonella *serogroups from cattle feces within the reference CAFO with C1 (53%) comprising the majority of the cattle fecal isolates. We isolated three *Salmonella *serogroups from cattle water troughs within the starling-controlled CAFO with C1 (45%) and E (45%) comprising the majority of the water trough isolates. We isolated three *Salmonella *serogroups from cattle water troughs within the reference CAFO with C1 (53%) comprising the majority of the water trough isolates. *Salmonella *serogroups E (60%) and C1 (40%) were isolated from feed within the starling-controlled CAFO. *Salmonella *serogroups E (75%) and C1 (25%) were isolated from feed within the reference CAFO.

**Table 1:** *Salmonella *serogroups by sample type

Serogroups (% of *Salmonella *positive samples)				
**Rank**	**Cattle feed bunks**	**Cattle water troughs**	**Cattle feces**	**Total**

1	E (66.6)	C1 (48.0)	C1 (46.4)	C1 (46.1)
2	C1 (33.3)	E (34.0)	E (39.1)	E (39.1)
3		C1 & E (8.0)	B (8.7)	B (4.7)
4		No serogroup data (6.0)	C2 (2.9)	No serogroup data (3.9)
5		C2 (4.0)	No serogroup data (2.9)	C1 & E (3.1)
6				C2 (3.1)
Total no.	9	50	69	128

From all sample types within both CAFO's we isolated a total of 13 different *Salmonella *serotypes with S. Montevideo (41%) and S. Anatum (26%) comprising the majority isolated (Table 2). Comparisons between CAFO's suggested there was little difference in the serotypes isolated. We isolated eight *Salmonella *serotypes from cattle feces within the starling-controlled CAFO with S. Montevideo (22%), S. Anatum (17%) and S. Agona (11%) comprising half of the serotypes isolated. Within the reference CAFO, we isolated eight *Salmonella *serotypes from cattle feces with S. Montevideo (49%), and S. Anatum (19%) comprising a majority of the serotypes isolated. We isolated four *Salmonella *serotypes from cattle water troughs within the starling-controlled CAFO with S. Anatum (50%) and S. Montevideo (20%) comprising a majority of the serotypes isolated. Within the reference CAFO, we isolated six *Salmonella *serotypes from cattle water troughs with S. Montevideo (47%) and S. Anatum (27%) comprising a majority of the serotypes isolated. *Salmonella *serotypes S. Meleagridis (60%) and S. Montevideo (40%) were isolated from cattle feed within the starling-controlled CAFO. *Salmonella *serotypes S. Anatum (50%), S. Montevideo (25%), and rough O: *e, h*: 1, 6 (25%) were isolated from cattle feed within the reference CAFO.

**Table 2:** *Salmonella *serotypes by sample type

Serotype (% of *Salmonella *positive samples)				
**Rank**	**Cattle feed bunks**	**Cattle water troughs**	**Cattle feces**	**Total**

1	Montevideo (33.3)	Montevideo (36.0)	Montevideo (42.0)	Montevideo (40.6)
2	Meleagridis (33.3)	Anatum (36.0)	Anatum (18.8)	Anatum (25.7)
3	Anatum (22.2)	Rough O: e, h: 1,6 (6.0)	Not serotyped (15.9)	Not serotyped (10.9)
4	Rough O: e, h: 1,6 (11.1)	Not serotyped (6.0)	Agona (7.2)	Rough O: e, h: 1,6 (4.6)
5		Lille (4.0)	Rough O: e, h: 1,6 (4.3)	Agona (3.9)
6		Multiple serotypes (4.0)	Multiple serotypes (4.3)	Multiple serotypes (3.9)
7		Meunchen (2.0)	Kentucky (1.5)	Meleagridis (3.1)
8		Meleagridis (2.0)	Kiambu (1.5)	Lille (1.6)
9		Newport (2.0)	Lexington_var._15+ (1.5)	Newport (1.6)
10		3,10: nonmotile (2.0)	Newport (1.5)	3,10: nonmotile (0.7)
11			Rough O: gms:- (1.5)	Kentucky (0.7)
12				Kiambu (0.7)
13				Lexington_var._15+ (0.7)
14				Meunchen (0.7)
15				Rough O: gms:- (0.7)
Total no.	9	50	69	128

## Discussion

We conducted this study to see if starling control could potentially be a viable management option for reducing *S. enterica *within CAFO's. Within the starling-controlled CAFO, detection of *S. enterica *disappeared from feed bunks and substantially declined within water troughs following starling control operations. Since contamination of cattle feed and water are obvious routes for the ingestion of *S. enterica*, starling control shows promise as a potential tool to help CAFO operations reduce *S. enterica *contamination. This relationship was not as clear for cattle fecal samples. During the pre-treatment through post treatment periods, herd prevalence of *S. enterica *increased on the reference facility but herd prevalence of *S. enterica *on the starling-controlled CAFO stayed at pretreatment levels.

Post control sampling may have occurred too early to detect *S. enterica *reductions within cattle fecal samples. Since the first of two DRC-1339 applications occurred a full two weeks before post control sampling we believed a change in fecal shedding rates could be detected within the starling-controlled pen lanes. In retrospect it is possible this time period was not long enough to detect a decrease in the number of cattle shedding *S. enterica*. Additionally, cattle fecal shedding of *S. enterica *is likely to be compounded by additional factors that contribute to the infection process.

The interactions among *S. enterica*, affected cattle and their environment are complex [5], and the role contaminated animal feed plays in *S. enterica *infections of food animals is not well understood [24]. This suggests that clinical and subclinical *S. enterica *infections in cattle are influenced by additional factors besides feed and water contamination. For example, herd size [35], age of cattle [35], manure management and disposal methods [25,36], feed storage [25], access to environmental waters [25], season [5], purchasing cattle from dealers [37], method of cattle penning [25], and exposure to wild birds and rodents [37,38] have all been implicated as herd-level risk factors for *S. enterica *infections. Thus, multiple biological, environmental and facility management factors will influence frequency and duration of cattle fecal shedding of *S. enterica*.

Based upon our data and previously published information we believe starling control should only be considered as part of a comprehensive disease management plan, not a stand alone tool to reduce *S. enterica *in CAFO's. In addition, starling control should only be used to manage *S. enterica *if additional research corroborates our findings. One should not assume this means starling control will not reduce *S. enterica *fecal shedding by cattle. If starling control is shown to be a reliable tool to reduce *S. enterica *loads in cattle feed and water, then starling control may have benefits throughout the farm to fork chain of food production. Reducing *S. enterica *ingested through feed and water supplies may help reduce the number of colony forming units shed within the feces of infected cattle. The dose of *S. enterica *ingested by cattle is known to influence the risk of clinical infections and the subsequent amount of fecal shedding [39,40]. Both contribute to carcass contamination within meat packing facilities which contributes to the contamination of human food products [11,12]. Thus, starling control may be a cheap and effective tool producers can add to existing strategies for managing disease while also reducing other negative economic impacts imposed by large numbers of starlings, such as feed loss and cleanup costs.

It is important to remember that the inference of our study was limited to the two CAFO's we sampled. Based upon our data we believe further research examining the efficacy of bird control as a tool to reduce the amplification and spread of disease in CAFO's is necessary. Studies examining the efficacy of starling control need to be replicated using multiple CAFO's in different geographic regions before control operations are adopted as a reliable disease management tool. Future research should also consider additional risks that could be attributed to starling use of CAFO's. For example, CAFO's have been implicated as potential sources for microbial pollution of the environment [41-43] thus starling control may help reduce the spread of *S. enterica*. LeJeune et al. [22] found that radio-collared starlings regularly traveled 20-km from their roost site to access dairies in Ohio and some of the radio collared starlings visited multiple facilities. This suggests that starlings could potentially transport *S. enterica *between CAFO's, aiding in the spread and maintenance of *S. enterica *between otherwise isolated facilities. Also, migratory starlings may transport *S. enterica *over large geographic areas. Hubálek [44] suggested that starlings could be one of many avian species responsible for wide ranging geographic dispersal of microorganisms. This is supported by the work of Palmgren [45] that found multidrug resistant strains of *S. enterica *Typhimurium in migrating birds in Sweden.

## Conclusion

The results of this study suggest that starling control may reduce the amplification and spread of *S. enterica *to cattle feed and water supplies. In addition, we also believe starling control may help reduce other chronic livestock diseases spread through the fecal-oral route of contamination. It is unlikely that the ecological interactions between European starlings, *S. enterica*, and cattle are the only disease risks that can be attributed to peridomestic wildlife use of CAFO's. Starlings may contribute to the maintenance and spread of other pathogens in CAFO's and other wildlife species may contribute to the maintenance and spread of *S. enterica*. Identifying high risk wildlife, pathogens, and their various ecological interactions with domesticated animals is needed to characterize the disease risks, production costs, and environmental impacts associated with peridomestic wildlife use of CAFO's.

## Methods

We conducted this study with the cooperation of two CAFO's located in Moore County, Texas, USA. Facilities were selected based on similarity of management practices and presence of starlings. Both facilities were large CAFO's; the starling-control CAFO had a herd size of 50,000 head of cattle and the reference CAFO had a herd size of 70,000 head of cattle. Neither facility raised other livestock and each was experiencing severe problems with starlings (> 10,000 starlings/day). Both facilities group housed approximately 100 to 150 mixed breed cattle per pen, and fed cattle finishing rations consisting of 75% steam flaked corn and 25% corn silage. Both facilities used auto filled open watering troughs fed from ground water that were cleaned 2 times a week. Feed bunks and water troughs were the only sources of food and water available to cattle. Prophylactic vaccinations were provided to newly acquired cattle at both facilities and antibiotics were provided in feed to manage disease in the herds. Manure was cleaned from pens 2 times per month using front-end loaders and dump trucks.

We estimated the number of starlings within pen lanes prior to sample collection. Pen lanes consisted of long rows of multiple interconnected pens. Roads separated pen lanes and no two lanes were interconnected. All cattle feed, water, and cattle feces within pen lanes were separated from the feed, water, and cattle feces in the other lanes.

Within individual pen lanes, cattle feces intermix between pens and cattle in adjoining pens have direct contact with each other through dividing fences. Some water troughs could be accessed from multiple pens and there were no dividers separating the feed between adjacent pens. Thus, we treated pen lanes as isolated islands of cattle within feedlots. Pen lanes differed in number of interconnected pens, and because pen lanes would vary in size, only the first 10 pens within each lane were sampled for starlings and *S. enterica*.

Five pen lanes were randomly selected for sampling from both the bird-controlled (DRC-1339 treated CAFO) and reference (no bird control operations) facilities. Lanes were randomly selected by drawing index cards from a garbage bag containing the lane numbers for each respective facility. All selected pen lanes within CAFO's were sampled before (18 January through 21 January) and after (15 February through 18 February) starling control operations.

Starling control operations were conducted by biologists from U.S. Department of Agriculture/APHIS/Wildlife Services, the Federal agency with responsibility for managing conflicts with wildlife [29]. Starling control was conducted using only approved methods that conform to the guidelines laid out in the 2000 report of the American Veterinary Medical Association Panel on Euthanasia [30] and set forth as agency policy in USDA/APHIS/WS Directive 2.505.

Wildlife Services biologists baited starlings using a 2% solution of DRC-1339 (3-chloro-p-toluidine hydrochloride) on treated corn chop. Technical DRC-1339 powder was mixed with water to create a 2% solution. Treated corn chop was soaked in the 2% solution and screen dried. The bait was applied at a concentration of 1:10 treated to untreated corn chop. All DRC-1339 applications were implemented in accordance with label requirements "Compound DRC-1339 Concentrate - Feedlots"; (EPA Registration 56228-10).

On 30 January and 9 February 2010, 1100 pounds of 1:10 treated corn chop was applied by hand or with the use of a modified trip hopper machine. Both hand and machine applications applied 1 pound/1000 ft^2 ^outside the south facing feeder bunks in the feeder truck lanes. Biologists timed the applications of DRC-1339 to immediately follow winter snow events because these conditions prevented starlings from loafing and feeding within cattle pens. The south-facing feed bunks created a wind-break with reflective sun warmth that protected starlings from cold northerly winds. Timing applications with ideal environmental conditions helped attract starlings to the bait lanes, increased bait consumption and target efficacy. We waited one week following the last DRC-1339 application to collect post-control *S. enterica *data. We selected one week because of the short time interval between *S. enterica *exposure and the onset of fecal shedding [31].

At both the starling-controlled and reference CAFO's we collected a total of 120 cattle feed, 120 cattle water samples and 120 cattle fecal samples. All samples were collected within 5 pen lanes. The same 5 pen lanes were sampled before and after starling control operations. Sample collection occurred four days before and four days after starling control operations. We collected three feed samples/pen lane/day and placed them into sterile Whirl-Paks^®^, and three 100 ml water samples/pen lane/day and placed them into sterile 125 ml plastic vials. We collected cattle fecal samples only when an animal was observed defecating. This eliminated cross contamination from other fecal pats and assured that the fecal sample was from a single cow. This process allowed us to estimate the percent of cattle shedding *S. enterica*. Three fecal samples were collected/pen lane/day and placed in sterile Whirl-Paks^®^. We did not sample from the same pen twice on the same day. All samples were immediately stored at 4°C and express shipped on the day of collection to the Colorado State University, Veterinary Diagnostic Laboratory (CSUVDL) in Fort Collins, Colorado for diagnostic testing.

We used standard operating procedures from CSUVDL for *Salmonella *culture. Briefly, ten-fold dilutions were made of each environmental sample type (10 g feed, 25 ml water) in pre-enrichment broth (buffered peptone water, Difco) and incubated overnight at 35°C. After pre-enrichment, 1 ml of the culture suspension was added to 10 ml of tetrathionate broth (Difco) and incubated overnight at 35°C [32]. Fecal samples were added at ten-fold dilutions to tetrathionate (Difco) broth and incubated overnight at 35°C [32]. For each sample type, 100 μL of the incubated tetrathionate suspension was transferred to 10 ml of Rappaport-Vassiliadis broth (Oxoid, Ogdensburg, NY) and incubated overnight at 42°C. A swab of the culture suspension was plated for isolation on brilliant green agar (Difco) and an XLT4 agar plate (BBL) and incubated for 24 hours at 35°C. Up to three suspect colonies based on colony morphology were picked and plated to blood agar plates. Following overnight incubation at 35°C, colonies were tested with polyvalent O-grouping antisera for agglutination. We shipped all positive samples to the National Veterinary Services Laboratory (NVSL) in Aims, Iowa for serotyping.

Our Before-After Control-Impact (BACI) study design [34] was analyzed using a two-factor repeated measures Analysis of Variance framework to address the research question: did *Salmonella enterica *in cattle feed, cattle water and cattle feces decrease in the starling-controlled CAFO relative to the reference CAFO? Data on *S. enterica *in cattle feed, water, and feces were analyzed separately as mixed linear models using SAS software. Fixed effects included sampling period (before and after DRC-1339 applications) and sites (DRC-1339 treated and reference CAFO's) and the interaction between sampling periods and sites. The pen lanes within CAFO's formed the experimental units upon which repeated observations were made.

This research project was reviewed and approved by the National Wildlife Research Center's (NWRC) Internal Animal Care and Use Committee (IACUC) prior to any data collection.

## Authors' contributions

JC contributed to the design of the study, conducted the field work and contributed to the statistical analysis and manuscript preparation. RE contributed to the design of the study, statistical analysis, and manuscript preparation. DH conducted the lab work and contributed to the manuscript preparation. RG conducted the starling control operations and contributed to the manuscript preparation. LC, TD and MB provided the financial and material support to conduct this study and contributed to manuscript preparation. GL is the project leader for our research group he oversaw the implementation of this research and contributed to the manuscript preparation.
